# Chronological changes of acquired agminated melanocytic nevi on the sole: A 9-year follow-up case report

**DOI:** 10.1016/j.jdcr.2026.05.048

**Published:** 2026-05-27

**Authors:** Akari Terada, Daiki Rokunohe, Daisuke Sawamura, Eijiro Akasaka

**Affiliations:** Department of Dermatology, Hirosaki University Graduate School of Medicine, Hirosaki, Japan

**Keywords:** acquired agminated melanocytic nevi, acral melanoma, chronology, East Asian, mosaic

## Introduction

Acquired agminated melanocytic nevi (AAMN) is a clustered group of melanocytic nevi localized to a limited area of the body.^1^ AAMN in the acral region is rare and can mimic melanoma due to its variegated appearance and the progressive increase in pigmented spots. In this report, we present a case of AAMN on the sole with a chronological overview of the lesion. While several cases of acral AAMN have been reported, detailed photographic documentation of the chronological evolution over such an extended period (9 years) has not been previously published.

## Case report

**Fig 1:**
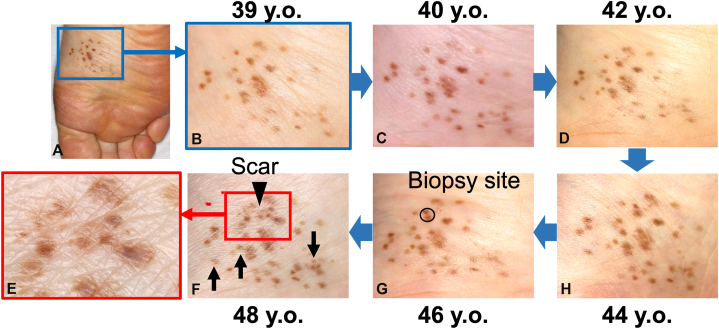
Clinical features of a case of acquired agminated melanocytic nevi. Brown pigmented macules on the right sole at 39 **(A** and **B)**, 40 **(C)**, 42 **(D)**, 44 **(E)**, 46 **(F)**, and 48 **(G)** years of age. Pigmented spots developed during the observation period, partially overlapping with preexisting spots **(G**, *black arrows***)**. The *circle* indicates the site of the skin biopsy **(F)**. Multiple pigmented spots developed predominantly around the biopsy scar **(G** and **H)**. The *arrowhead* indicates the scar.

**Fig 2:**
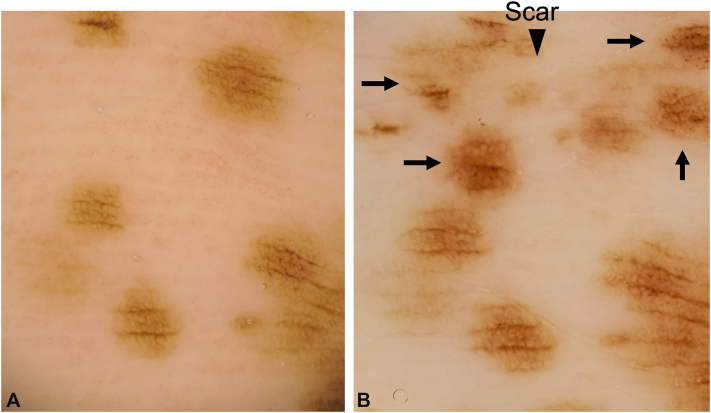
Dermoscopic examination revealed parallel furrow patterns at 39 **(A)** and 48 **(B)** years of age. During the 9-year follow-up, some spots became darker, and new spots emerged, particularly around the biopsy scar (*black arrows*).

A 39-year-old woman presented to our department with multiple small pigmented lesions on her right sole. She reported that the initial 12 pigmented spots were not present at birth but appeared at age 12. The number of pigmented spots began to increase spontaneously when she reached the age of 36, with no identifiable triggers. At 39 years of age, she became concerned and was referred to our hospital. Clinical examination revealed multiple brown pigmented spots (1-5 mm in diameter) clustered on the right sole. The spots were confined to a limited 3-cm diameter area, forming a color-variegated region (Fig 1, *A* and *B*). There were no visible background pigmented macules, such as those seen in nevus spilus. Dermoscopic examination revealed that each spot exhibited parallel furrow patterns (PFPs) (Fig 2, *A*). Based on these findings, she was clinically diagnosed with AAMN and has been followed periodically.

During the 9-year follow-up (Fig 1, *B*-*G*), some spots became darker, and new spots emerged (Figs 1, *G* and 2, *B*, black arrows). In all lesions, including those not presented in the figures, dermoscopy consistently revealed PFP. We observed that newly emerged macules initially appeared as faint lines corresponding to the skin furrows, with pigmentation subsequently spreading around them (Fig 2, *B*). Throughout the 9-year follow-up, although the number of spots increased within the localized area, the size of individual spots remained small and relatively uniform without expanding beyond a specific range. No parallel ridge patterns or irregular fibrillar patterns were observed. Although individual spots consistently exhibited PFP, evaluating the entire cluster as a single lesion using the revised 3-step dermoscopic algorithm^2^ revealed atypical features that did not fulfill the criteria for a benign pattern. This diagnostic ambiguity prompted us to perform a skin biopsy to confirm the histopathology when the patient was 46 (Fig 1, *F*).

**Fig 3:**
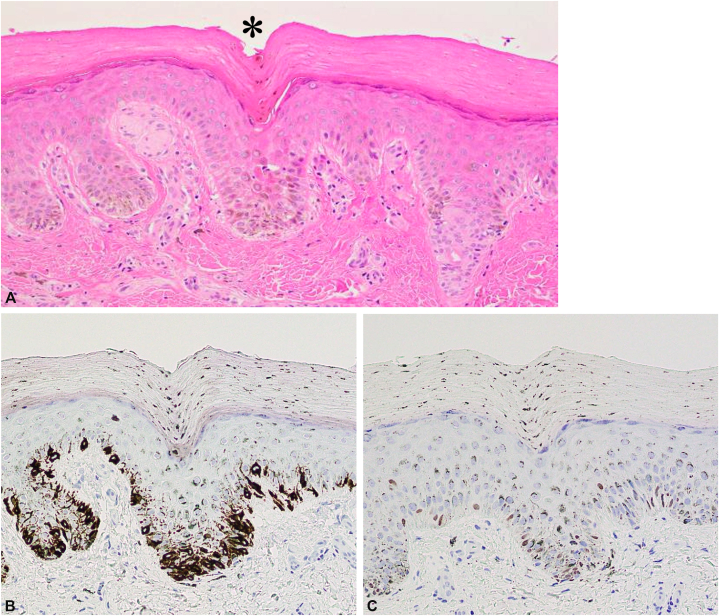
Histopathologic analysis showed melanocytic components near the dermal-epidermal junction, predominantly arranged at the tips of the rete ridges. Melanin deposition was observed in the stratum corneum, corresponding to a plantar furrow **(A**, *asterisk*, hematoxylin and eosin stain**)**. Immunohistochemical staining for Melan-A **(B)** and PRAME **(C)**. Original magnification ×200.

Histopathologic analysis revealed melanocytic proliferation near the dermal-epidermal junction, predominantly arranged at the tips of the rete ridges (Fig 3, *A*). The melanocytes were round and exhibited no nuclear atypia. Immunohistochemistry for Melan-A and preferentially expressed antigen in melanoma (PRAME) was performed to differentiate AAMN from melanoma (Fig 3, *B* and *C*). Although Melan-A-positive cells were densely distributed in the basal layer, only a few cells were positive for PRAME. The nuclei of PRAME-positive cells showed no atypia. PRAME is reportedly positive in 94.4% of acral melanomas.^3^ Notably, the same study also reported that 13.6% of benign nevi can exhibit PRAME positivity.^3^ The sparse PRAME positivity and the lack of nuclear atypia in our case support a diagnosis of benign nevi. There was no remarkable increase in the number of melanocytes or nevus cells in either the epidermis or dermis surrounding the pigmented lesion. Two years after the biopsy, multiple pigmented spots developed predominantly around the biopsy scar (Fig 1, *G* and *H*). Throughout the follow-up period, the patient showed no evidence of melanoma arising from AAMN.

## Discussion

Torchia reviewed 19 cases of common acquired melanocytic nevi grouped on normal background skin and found no significant site predilection.^1^ Including our case, six cases of AAMN on the sole have been reported in international journals,^4^, ^5^, ^6^ 5 of which were from Japan. In contrast, only a few cases of AAMN in other body regions have been reported from Japan, suggesting that AAMN on the sole may be more common among Japanese individuals. Because acral melanoma is the most prevalent type of melanoma in East Asians,^7^^,^^8^ careful differentiation of acral AAMN from melanoma is essential.

AAMN is regarded as a phenotypical pattern of mosaicism.^1^ While pigmentary mosaicism typically follows Blaschko's lines on the trunk, the distribution pattern on acral regions remains less characterized in the literature. In our case, several new pigmented spots developed over the 9-year follow-up, and all were strictly localized within a 3 cm oval area on the sole rather than following linear or segmental patterns. Previous cases also showed a similar distribution pattern of pigmented lesions,^6^ which may reflect unique features of cutaneous mosaicism in acral regions. Luo et al^9^ reported a case of agminated segmental nevi where the Braf^V600E^ mutation was detected exclusively within the pigmented lesions, supporting the theory of a mosaic condition.

Another interesting finding was that new pigmented spots were predominantly clustered around the biopsy scar, whereas only a few were scattered in other areas. This uneven distribution of new lesions raises the possibility that posttraumatic inflammation or mechanical stimulation may contribute to the development of these lesions, although the underlying pathogenetic mechanisms remain speculative. Navarini et al^10^ described a case of eruptive nevi triggered by localized superficial trauma. Their study discusses the potential role of cytokines and cellular interactions between melanocytes and other cells, such as keratinocytes and fibroblasts, in the development of posttraumatic AAMN. The role of external stimuli in AAMN progression warrants further investigation. Furthermore, it remains unclear whether the progressive development of AAMN is specific to acral regions or occurs similarly in other anatomical sites, because long-term follow-up data are limited. Further accumulation of cases is needed to elucidate site-specific differences in AAMN behavior.

Although molecular analyses were not performed in our case, future studies incorporating genetic analyses could provide valuable insights into the somatic mutations underlying AAMN.

### Declaration of generative AI and AI-assisted technologies in the writing process

During the preparation of this work, the authors used Claude Sonnet 4.5 in order to improve language. After using this tool, the authors reviewed and edited the content as needed and take full responsibility for the content of the published article.

## Conflicts of interest

None disclosed.
